# Impacts of Internet-Based Interventions for Veterans With PTSD: A Systematic Review and Meta-Analysis

**DOI:** 10.3389/fpsyg.2021.711652

**Published:** 2021-11-24

**Authors:** Yang Zhou, Zhenggang Bai, Wei Wu, Zijia Fan, Cuiying Wu, Longyi Li, Siyu Li

**Affiliations:** Department of Sociology, School of Public Affairs, Nanjing University of Science and Technology, Nanjing, China

**Keywords:** meta-analysis, veterans, PTSD, internet-based intervention, randomized controlled trials

## Abstract

**Background:** Veterans who did not seek and complete treatment as intended have been shown to have an elevated risk of experiencing and being exposed to post-traumatic stress disorder (PTSD). Internet-based interventions (IBIs) provide more confidentiality and fewer treatment barriers, and they are regarded as potential treatments to reduce PTSD in veterans. However, the effects of IBI for veterans with PTSD are inconclusive.

**Objectives:** IBI is defined as any internet-based series of psychosocial interventions, of which the internet works as a way of delivery. Psychosocial content and reduction of PTSD symptoms in veterans have been recognized as two core elements of this intervention. This study aimed to (1) examine the effects of IBI on veterans’ PTSD outcomes and (2) distinguish between the elements of IBI that play an important role for veterans with PTSD.

**Methods:** Web of Science, PubMed, EMBASE, PsycINFO, Cochrane, Wanfang Data, CNKI, and CQVIP databases were searched for randomized controlled trials (RCT) in IBI programs for veterans with PTSD, covering all studies in English and Chinese published from January 1990 to November 2020. Also, related studies tracking citations were identified. Studies met the following inclusion criteria of (1) being RCTs; (2) containing IBI in the full text; (3) having IBI conducted on veterans as participants; and (4) being on PTSD. All processes followed PRISMA. The risk of bias of the studies was assessed by the Cochrane Systematic Review Handbook. The confidence of outcomes of this review was valued according to the GRADE (Grading of Recommendations Assessment, Development, and Evaluation). The meta-analysis was done by RevMan 5.13. Two teams of reviewers independently searched the literature, made the assessment, and extracted the data.

**Results:** A total of 1,493 citations were identified after initial searching, of which the full texts of 66 studies were screened. Eventually, six RCT studies met the inclusion criteria. Beneficial effects of IBI were found on the overall PTSD outcome (−0.29; 95% CI–0.48 to −0.11, *p*<0.01). Particularly, IBI based on cognitive behavioral therapy (CBT) with peer support was found to be effective for PTSD outcomes (−0.36; 95% CI–0.61 to −0.11, *p*<0.01). The subgroup analysis demonstrated that scores of PTSD outcome measured by a PCL (PTSD Checklist) decreased to an average score of 0.38 (95% CI –0.60 to −0.15, *p*=0.001). The intervention had a positive effect on the PTSD outcome on veterans with comorbid psychological disorders (−0.30; 95% CI –0.61 to −0.11, *p*<0.01). Overall, the six studies included were evaluated with a low risk of bias, and the outcomes of the meta-analysis were proven with high confidence.

**Conclusion:** On the whole, IBIs have a positive effect on the overall PTSD outcome of veterans. The results encouraged us to focus on IBI with CBT with peer support for veterans, on specific instruments for veterans with PTSD, and on veterans with comorbid psychological disorders. This study, however, has limits. Only six studies with a Western population were included, which might result in cultural bias on IBI effects. In future, more high-qualified research and diverse cultural background of RCTs is needed to prove the effectiveness of IBI on veterans with PTSD.

## Introduction

Post-traumatic stress disorder (PTSD) is a prevalent, chronic, and disabling psychiatric disorder often diagnosed among military personnel in relation to combat trauma (Possemato et al., 2011; McLean and Foa, 2013). A combination of childhood abuse, combat traumas, and/or sexual assault was suggested as the reason for veterans’ PTSD (Morland et al., 2015). The study by Niemeyer et al. (2020) proved that PTSD had a 12-month prevalence rate of 3.7% among the United States population and 2.3% in the German population and other European countries. Among veterans of the wars in Iraq and Afghanistan, PTSD prevalence was estimated to be 10–20%, and female military personnel were more likely than male veterans to experience higher PTSD prevalence rates (Morland et al., 2015).

PTSD is related to many negative effects on patients, such as significant adverse health and life consequences (Morland et al., 2015). PTSD can cause severe distress, chronic suffering, and impairments, of which the core symptoms include persistent avoidance of traumatic content, re-experiencing traumatic content, and negative alterations in cognition, arousal, and reactivity (Haagen et al., 2015). Firstly, PTSD results in altered cognition that includes hypersensitivity to physical sensations or mislabeling these symptoms as common physical health problems (Possemato et al., 2011). Individuals with PTSD report poorer general health and bodily pain (Stevens et al., 2016; Hamblen et al., 2019). Also, veterans with PTSD often have high rates of comorbid mental health disorders (Engel et al., 2015). When patients do not receive timely and effective treatment, PTSD often becomes a chronic and disabling disorder that is often comorbid with substance abuse disorders, major depression, psychological distress, and other anxiety disorders (McLean and Foa, 2013; Niemeyer et al., 2020). What is worse, PTSD is associated with impairment in social/occupational functioning (Hamblen et al., 2019). Veterans with PTSD are believed to be less able to engage in physical activities or fulfill previously held roles (Stevens et al., 2016). They are reported to have increasing impairments in performing life roles (e.g., work and family responsibilities) and daily tasks (e.g., household chores; Stevens et al., 2016). PTSD is also related to violent behavior (e.g., aggression; Morland et al., 2015).

Traditionally, several evidence-based psychotherapeutic approaches to treat PTSD have been recommended, including CBT, cognitive processing therapy (CPT), prolonged exposure therapy (PE), stress management skills training, eye movement desensitization reprocessing (EMDR), and other innovative interventions (Kearney et al., 2012; Rodriguez et al., 2020). Cognitive-behavioral conjoint therapy (CBCT) and integrative behavioral couple therapy (IBCT) were designed to combine acceptance strategies and IBCT techniques to ameliorate the symptoms of PTSD, relationship conflicts, and experiential avoidance (Monson et al., 2009). Structured approach therapy (SAT) as an approach in a stress inoculation therapy (SIT) framework consists of psycho-education, skills training, and coping skills (Sautter et al., 2015). PE has also been shown effective in lessening PTSD symptoms and maintaining treatment gains over time (McLean and Foa, 2013). EMDR produced significant enhancements in decreasing anxiety, anger, depression, isolation, and other symptoms (Koven, 2018). Mindfulness-based stress reduction (MBSR) is a standardized class series to teach mindfulness to reduce stress by a combination of meditation, body awareness, and mindful breathing, which have been shown to have significant improvements in PTSD symptoms (Kearney et al., 2012; Colgan et al., 2016; Koven, 2018). Mindfulness-based cognitive therapy (MBCT) also belongs to stress-reduction groups involved psychotherapies incorporating mindfulness techniques (King et al., 2013). Yoga has also been used to manage stress *via* adjusting the cognitive perspective and focusing on inner peace (Reddy et al., 2014). Building spiritual strength (BSS) is an inter-faith, manualized, spiritually integrated intervention to support the participants in their choice of religious affiliation or non-affiliation (Harris et al., 2011). Logotherapy has also been applied as an innovative adjunctive treatment for PTSD by healing through meaning (Koven, 2018). Another complementary intervention is a specially trained psychiatric service dog, offering emotional and therapeutic value (Rodriguez et al., 2020).

Traditional intervention sessions are usually conducted by therapists with the veteran and the veteran’s partner (Sautter et al., 2015). While, compared with non-military groups, veterans have a higher need for services addressing PTSD. Perceived stigma of treatment seeking was proven to be negatively related to treatment utilization in a study with a sample of 812 veterans (Kulesza et al., 2015). And for some veterans, logistics and confidentiality concerns also posed problems with treatment utilization (Engel et al., 2015; Krupnick et al., 2017; Niemeyer et al., 2020) Nontraditional online health care pathways have been proposed as possible solutions to cope with the disadvantages of stigma, confidentiality concerns and treatment inefficacy (Parish et al., 2014). It was found that web-based interventions for PTSD were a feasible and helpful intervention for veterans (Belsher et al., 2015). The feasibility, acceptability, and usability of IBI have been well documented (Bush et al., 2011).

According to Barack and Klein, IBIs are defined as therapeutic programs with various health objectives mainly delivered *via* the internet, described with respect to four major components: content, multimedia, interactive online activities, and guidance and supportive feedback (Barak et al., 2009; Guay et al., 2017). The U.S. Defense Department’s National Center for Telehealth & Technology has developed a web through which to provide a set of information on resources, tools, and aids for military personnel, veterans, and their families (Bush et al., 2011). Web-based self-management has been proved an effective online tool for the military (Bush et al., 2011). A web-based video gallery has been provided to share personal stories about PTSD and treatments (Hamblen et al., 2019).

In a systematic review and meta-analysis, e-Mental health interventions revealed a significant improvement in PTSD symptoms in the active intervention group among a general population (Simblett et al., 2017). Yet, to our knowledge, there is limited literature on meta-analysis and systematic review of IBI on veterans with PTSD. One review analyzed the prevalence of eight disorders among elderly veterans and found that PTSD prevalence was not markedly high (Williamson et al., 2018). To reduce PTSD symptoms, a meta-analysis suggested that iCBT for PTSD was efficacious, and only one study was analyzed based on a military sample (Niemeyer et al., 2020). One review included 12 studies to access the interventions for supporting partners of military veterans with PTSD, among which only one was based on IBI (Turgoose and Murphy, 2019). However, IBIs in other groups were tested as effective in supporting informal caregivers (Sherifali et al., 2018), which helped us to see the potential of IBI on veterans with PTSD in our study. Therefore, the primary aim of this research was to conduct a systematic review and meta-analysis to examine the effects of IBI on veterans diagnosed with PTSD who were living in a community or absent from military life. The secondary objective was to distinguish the contents of IBI that played an important role for veterans to decrease levels of PTSD.

## Materials and Methods

### Population

A total of 622 participants in all six studies included veterans, members of the active military on leave with PTSD, and a small number of civilians diagnosed with PTSD (see Table 1). The average age of the participants in the studies was 38.88.

**Table 1:** Key characteristics of participants and RCTs in the studies.

Study	Participants	Country	Comorbidity	Treatment type		N1	Experimental group			N2	Control group			Outcome measure
				Control group	Experimental group		Mean age	Age range	Sex (M/F)		Mean age	Age range	Sex (M/F)	
Engel et al., 2015	Service members and veterans	United States	Depression and anxiety	OUC^e^	DESTRESS-PC^e^	43	36.2	28.45–43.95	34/9	37	36.7	26.95–46.45	31/6	PCL^d^
Morland et al., 2015	Female civilian and military	United States	Depression, anxiety and substance use disorder	NP^e^	VTC^e^	63	46.9	35.1–58.7	0/63	63	46.0	33.9–58.1	0/63	CAPS^d^
Niemeyer et al., 2020	Male veterans and military	Germany	Depression, anxiety and dysthymia	-	iCBT^e^	21	37.7	30.84–44.56	21/0	17	37.8	25–50.6	17/0	CAPS
Litz et al., 2007	Service members	United States	Depression and anxiety	Internet-based supportive counseling	Internet-based, self-management cognitive behavior therapy	24	38.63	29.22–48.04	18/6	21	39.86	32.14–47.58	17/4	PCL
Stevens et al., 2016	Veterans	United States	Depression and perceived physical health impairment	AAU^e^	VP^e^	209	34.36 ± 8.01^a^	20–63^b^	170/30	94	-	-	77/17	PCL-M^d^
Possemato et al., 2019	Veterans	United States	Hazardous alcohol use	Self-management thinking forward	Peer-supported thinking forward	15	39 ± 9^a^	NA^c^	14/1	15	-	-	14/1	CAPS

a Mean age of population in both experimental group and control group.

b Age range of population in both experimental group and control group.

c Not reported in included study.

d PCL: PTSD Checklist; CAPS: Clinician-Administered PTSD Scale; PCL-M: Checklist – Military Version.

e OUC: optimized usual primary care PTSD Treatment; DESTRESS-PC: Delivery of self-training and education for stressful situations online; NP: in person VTC: videotele conferencing; iCBT: online cognitive behavior therapy; AAU: Adjustment as Usual; VP: Vets Prevail.

### Interventions

In the definition of IBI, the internet works as a method of delivery assisted by therapists, mental health providers, counselors or specialists, and nurses. The platform aims at conducting PTSD care and relief for veterans by stress-management skills, cognitive reframing techniques, treatment sessions, and CBT lessons with peer support.

### Inclusion and Exclusion Criteria

Two teams of researchers screened the literature independently. Research studies must meet the following inclusion criteria: be RCTs; contain IBI in the full text; have IBI conducted on veterans in trial groups; have no intervention, a delayed intervention, or another psychosocial intervention or usual care but not online conducted on participants in the control group; have PTSD as the outcome; be studies published between January 1990 and November 2020; and be studies published in English or Chinese.

Interventions conducted in closed settings (e.g., medical institutions) or those including the use of drugs were excluded. To ensure the quality of the study, research not published in a peer-reviewed journal, without data extraction or of detailed information for meta-analysis was also excluded.

### Search Strategy

We conducted a detailed automated search and screened relevant literature in the Web of Science, PubMed, EMBASE, PsycINFO, Cochrane, Wanfang Data, CNKI, and CQVIP databases for articles that were RCT in IBI programs for veterans with PTSD. Also, reference lists of related studies were manually tracked. Our search term consisted of three subsets: participant (“veteran” and “military”), intervention (“internet” and “web”), and outcomes (“PTSD” and “mental health”). We connected them with two Boolean operators (AND and OR) to search for relevant research in English and in Chinese published from January 1990 to November 2020 from the databases.

### Selection Results

**Figure 1:**
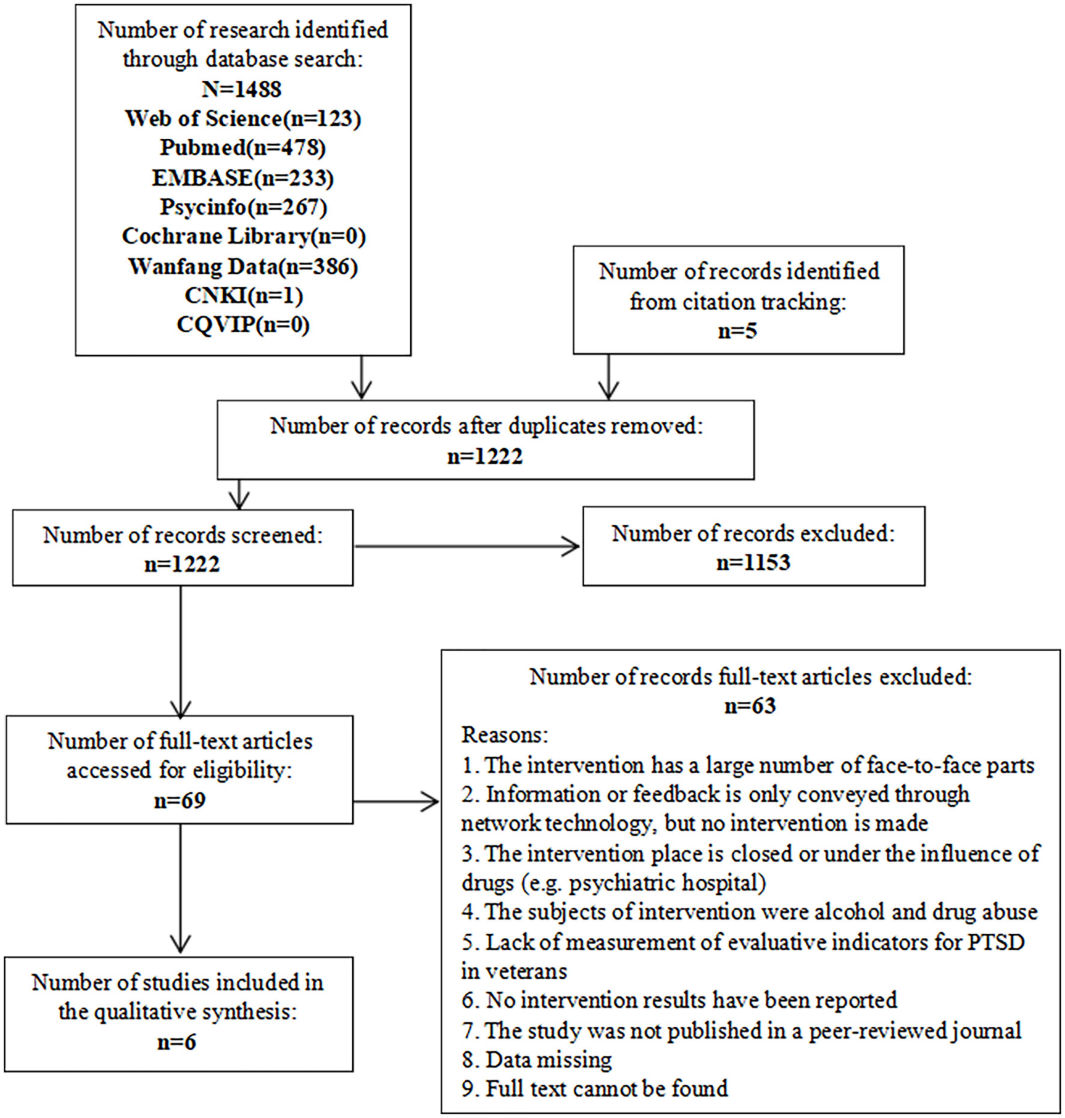
PRISMA diagram of included studies in the meta-analysis.

We followed the PRISMA statement to select studies (Moher, 2010). As shown in Figure 1, a total of 1,493 studies in the literature related to the topic were identified from eight databases (*n*=1,488) and other resources (*n*=5). After the removal of duplicate literature, 1,222 articles remained. Based on the inclusion and exclusion criteria, two teams of reviewers independently screened and excluded a total of 1,153 articles as non-related. The remaining 69 articles were further screened after reading the full-text articles. Considering that there might be topic-related articles or the titles and abstracts were insufficient to judge whether articles were useful. In the remaining 69 studies, we identified six eligible RCTs for our meta-analysis.

### Date Extractions

To answer the research questions, we generated a data extraction table. The coding scheme mainly extracted the contents, including participants’ characteristics, intervention design, outcome, measurement tool, and the duration of follow-ups. Basic information was extracted, including title, authors, country of authors, publication year, abstract, objective, and the journal. We also addressed the quantitative data: mean and standard deviation (SD). Some literature did not report these data, and we calculated the mean and SD of these studies according to the standard error (SE), *d* effect size, values of *p*, 95% confidence intervals (CIs), and other information given in the papers. The detailed information of these studies is reported in Tables 1–4.

**Table 2:** Summary of overall effectiveness of IBI on veterans with PTSD.

Author (year)	Intervention/Control	Estimate standard mean difference (95%CI)	*I*^2^ (%)	*p*	GRADE quality assessment
Engel et al., 2015	30/25	−0.20[−0.73, 0.34]	–	–	–
Litz et al., 2007	8/10	−0.89[−1.88, 0.09]	–	–	–
Morland et al., 2015	38/45	−0.14[−0.57, 0.30]	–	–	–
Niemeyer et al., 2020	21/17	−0.07[−0.71, 0.57]	–	–	–
Possemato et al., 2019	9/11	−0.10[−0.99, 0.78]	–	–	–
Stevens et al., 2016	166/90	−0.38[−0.64, −0.12]	–	–	–
Total	272/198	−0.29[−0.48, −0.11]	0	0.002	High

**Table 3:** Summary of subgroup analysis on intervention contents, outcome instruments, and comorbidity of participants.

Group	Subgroup	Number of studies	Intervention/Control	Estimate standard mean difference (95%CI)	*I*^2^ (%)	*p*	GRADE quality assessment
Intervention contents	Psycho-education	2	38/35	−0.35(−0.82 to 0.12)	33	0.14	High
	psychotherapy	2	59/62	−0.12(−0.47 to 0.24)	0	0.53	High
	CBT with peer support	2	175/101	−0.36(−0.61 to −0.11)	0	0.004	High
Outcome instruments	CAPS	3	68/73	−0.11(−0.45 to 0.22)	0	0.50	High
	PCL	3	204/125	−0.38(−0.60 to −0.15)	0	0.001	High
Comorbidity of participants	Depression and anxiety	5	263/187	−0.30(−0.49 to −0.11)	0	0.002	High
	Hazardous alcohol use	1	9/11	−0.10(−0.99 to 0.78)	/	0.82	High

**Table 4:** Summary of sensitivity analysis on sample size, gender and culture.

Subgroup	Number of studies	Intervention/Control	Estimate standard mean difference (95%CI)	*I*^2^ (%)	*p*	GRADE quality assessment
Exclude large sample size	5	106/108	−0.19[−0.46, 0.08]	0	0.16	High
Exclude female civilians and military	5	234/153	−0.33[−0.54, −0.12]	0	0.002	High
Exclude male veterans and military	5	251/181	−0.31[−0.51, −0.12]	0	0.002	High

All data extraction was completed by two independent reviewers and verified by another reviewer, to ensure the reliability of data extraction. The third reviewer was also responsible for the reconfirmation of inconsistent data that the two reviewers extracted. All reviewers agreed upon the content of the final data extraction table after discussion.

### Quality Assessment

Two independent reviewers assessed the risk of bias of each eligible study by the Cochrane Systematic Review Handbook. This quality assessment evaluates the levels of bias in the following content: selection, performance, detection, attrition, reporting, and other biases (Higgins et al., 2011). The confidence of outcomes of this review was also evaluated by two reviewers and rechecked by the third reviewer according to GRADE, which includes the risk of bias, inconsistency, indirectness, inaccuracy, and publication bias (Grade Working Group, 2004; Moher, 2010). All disagreements on risk bias and quality assessment were discussed to reach a conclusion among the reviewers.

### Data Analysis

We used the data (mean, SD) to perform the meta-analysis on the effectiveness of the IBI. *I*^2^ was used to demonstrate the heterogeneity among the studies in quantitative statistics and when the heterogeneity test *I*^2^<50%, a fixed-effects model, was used for meta-analysis (Higgins et al., 2003). Accordingly, this study used a fixed-effect model to explore the relationship between the studies. According to the differences in intervention content, measurement tools, and status of comorbidity, we grouped the studies and conducted a subgroup analysis to determine which components were playing key roles in mitigating PTSD symptoms in veterans. We also conducted a sensitivity analysis to test the stability of outcomes. The meta-analysis was done by RevMan 5.3.

## Results

### Description of Studies

As shown in Table 1, one of the studies was conducted in Germany (Niemeyer et al., 2020) and the other five in the United States. The mean age of the participants was under 50 (range 36.2–48). Two studies did not report the mean age of the experimental group and the control group (Stevens et al., 2016; Possemato et al., 2019). More than half the veterans were male in the majority of the studies (range 81.25–100%), except for one study in which all participants were female (Morland et al., 2015).

All six studies had experimental groups defined as veterans receiving IBI, and five studies had control groups with veterans having optimized usual primary care (OUC; Engel et al., 2015), treatment in person (NP; Morland et al., 2015), web-based supportive treatment (Litz et al., 2007; Possemato et al., 2019) and no treatment (Stevens et al., 2016; Niemeyer et al., 2020). One study had civilian women as participants in the control group (Morland et al., 2015). In all six studies, there were three types of intervention contents as branches of IBI. Two of these studies (33.33%) were categorized as using IBI in psycho-education, which contained self-training and education for stressful situations and self-management treatment (Litz et al., 2007; Engel et al., 2015). Two studies (33.33%) were regarded as using IBI in psychotherapy, which included two kinds of trauma-focused psychotherapy (Morland et al., 2015; Niemeyer et al., 2020). Two studies (33.33%) used IBI on CBT with peer support, which provided a platform for veterans to build social connections (Stevens et al., 2016; Possemato et al., 2019). PTSD outcomes were examined by three subgroups of the IBI, as shown in Table 3. Outcome assessment tools used for the outcome were categorized as CAPS and PCL. Three studies were assessed by CAPS (Morland et al., 2015; Possemato et al., 2019; Niemeyer et al., 2020), and the others were measured by PCL (Litz et al., 2007; Engel et al., 2015; Stevens et al., 2016). In total, five of the six studies were judged as small in sample size (≤100; Litz et al., 2007; Engel et al., 2015; Morland et al., 2015; Possemato et al., 2019; Niemeyer et al., 2020), and one study contained a large sample with 209 recipients in the experimental group and 94 recipients in the control group (Stevens et al., 2016). The length of follow-up in all studies was short (≤6months). Regarding the recipients’ mental health condition, six studies included veterans with PTSD (Litz et al., 2007; Engel et al., 2015; Morland et al., 2015; Stevens et al., 2016; Possemato et al., 2019; Niemeyer et al., 2020). Varying degrees of psychological disorders (depression, anxiety, dysthymia, substance use disorder, and perceived physical health impairment) were represented as comorbidities in five studies (Litz et al., 2007; Engel et al., 2015; Morland et al., 2015; Stevens et al., 2016; Niemeyer et al., 2020). In one study, veterans struggled with hazardous alcohol use in addition to PTSD (Possemato et al., 2019).

### Descriptions of Risk of Bias

**Figure 2:**
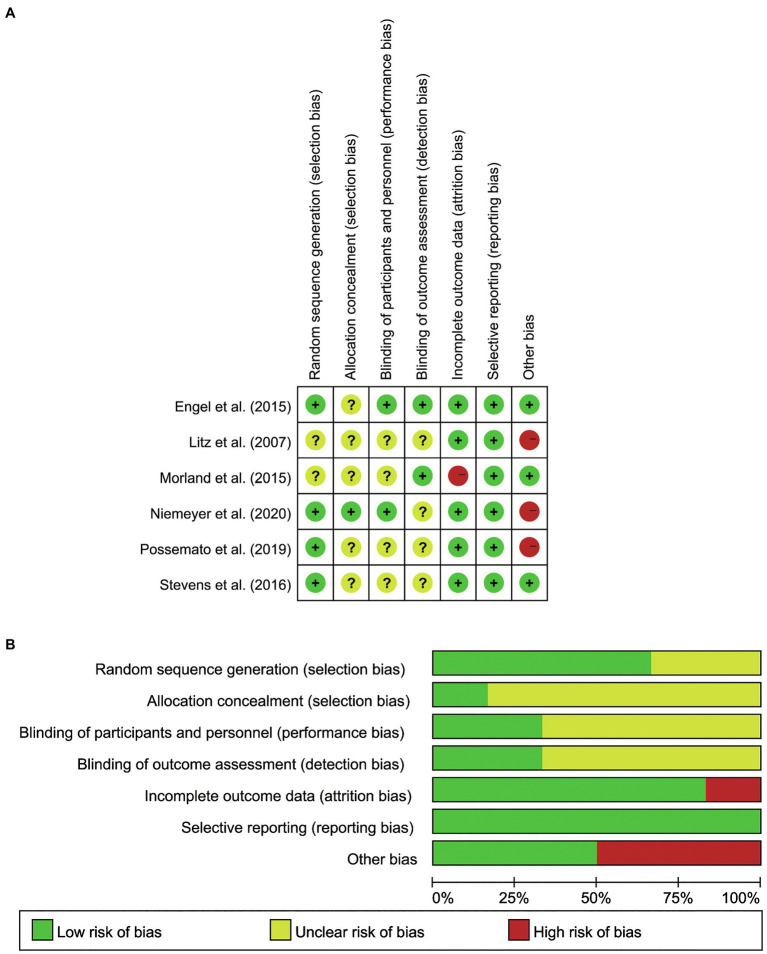
**(A)** Summary of Risk of Bias for all the included studies. **(B)** Graph of Risk of Bias for all the included studies.

The Cochrane Risk of Bias reported the quality of study methodology. The results of the critical assessments of the studies for the risk of bias on aspects of Allocation Assignment Methods, Allocation Concealment, Blinding of Participants Providers, Blinding of Outcome Assessment, Completeness of Outcome Data, Selective Reporting, and Other Bias are shown in Figures 2A,B. In Allocation Assignment Methods, four studies (66.67%) were low-risk (e.g., Engel et al., 2015), and two studies were unclear (33.33%; e.g., Litz et al., 2007). In regard to Allocation Concealment, one study (16.67%) was low-risk (Niemeyer et al., 2020), and five studies (83.33%) were unclear (e.g., Engel et al., 2015). For Blinding of Participants Providers, two studies (33.33%) were low-risk (e.g., Engel et al., 2015), and four studies (66.67%) were unclear (e.g., Stevens et al., 2016). For Blinding of Outcome Assessment, two studies (33.33%) were low-risk (e.g., Engel et al., 2015), and four (66.67%) were unclear (e.g., Stevens et al., 2016). With respect to Completeness of Outcome Data, five studies (83.33%) were low-risk (e.g., Engel et al., 2015), and only one study (16.67%) was high-risk (Morland et al., 2015). On Selective Reporting, all six studies (100%) were low-risk. In Other Bias, three studies (50%) were low-risk (e.g., Engel et al., 2015), and three studies (50%) were high-risk (e.g., Niemeyer et al., 2020).

### Results of Overall Effectiveness

**Figure 3:**
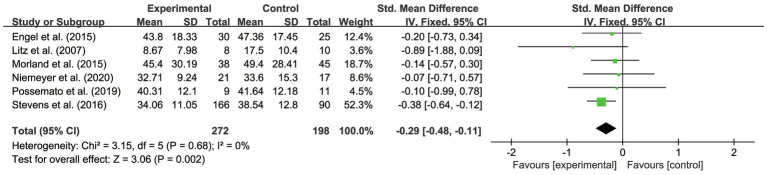
Forest plot of overall effectiveness of IBI on veterans with PTSD.

This systematic review and meta-analysis examined the overall effects of IBI on veterans’ PTSD outcomes. As shown in Table 2 and Figure 3, the scores dropped an average of 0.29 (95% CI –0.48 to −0.11, *p*<0.01). It was also found that there was no heterogeneity between the studies (*p*<0.01; *I*^2^ =0%). The assessment was evaluated as having high quality.

### Results of Subgroup Analysis

**Figure 4:**
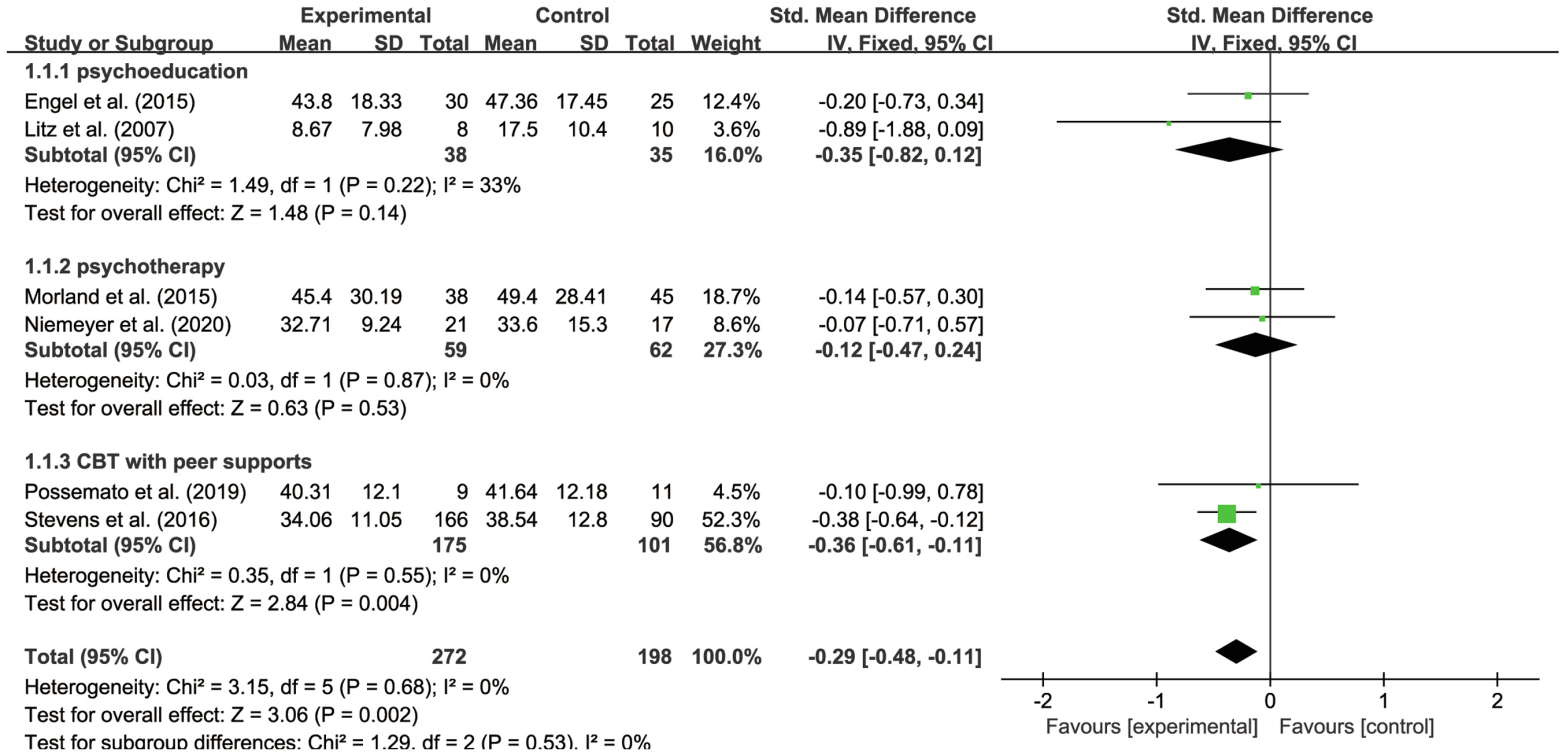
Forest plot of effects of IBI based on various contents.

For the subgroup of intervention measures, three kinds of IBI content have been distinguished. All six studies applied mature IBI training platforms or treatments: online self-management training (Engel et al., 2015), psycho-education *via* the internet (Litz et al., 2007), online therapy (iCBT; Niemeyer et al., 2020), videotele conferencing (VTC; Morland et al., 2015), CBT with a forum (Stevens et al., 2016), and CBT with peer support (Possemato et al., 2019). Considering the content and targets of treatments, we identified three subgroups of these interventions, which focused on providing psycho-education (Litz et al., 2007; Engel et al., 2015), psychotherapy (Morland et al., 2015; Niemeyer et al., 2020), and CBT with peer support (Stevens et al., 2016; Possemato et al., 2019). As suggested in Table 3, we found that for PTSD symptoms of veterans, the effects of CBT with peer support (SMD=−0.36, 95% CI −0.61 to −0.11) were more helpful than were the effects of psycho-education (SMD=−0.35, 95% CI −0.82 to 0.12) and the influences of psychotherapy (SMD=−0.12, 95% CI −0.47 to 0.24). According to Figure 4 (see Appendix 1), it was also found that the subgroups were not significantly different (*p*>0.05; *I*^2^=0%), suggesting there was no heterogeneity between the groups. Yet, the result with IBI based on psychotherapy has been found significantly effective on PTSD outcome (*p*<0.01). IBI with psycho-education (*p*>0.05) and peer support (*p*>0.05) had no statistically significant effects on veterans’ PTSD outcomes. The results show high quality evaluated by GRADE.

**Figure 5:**
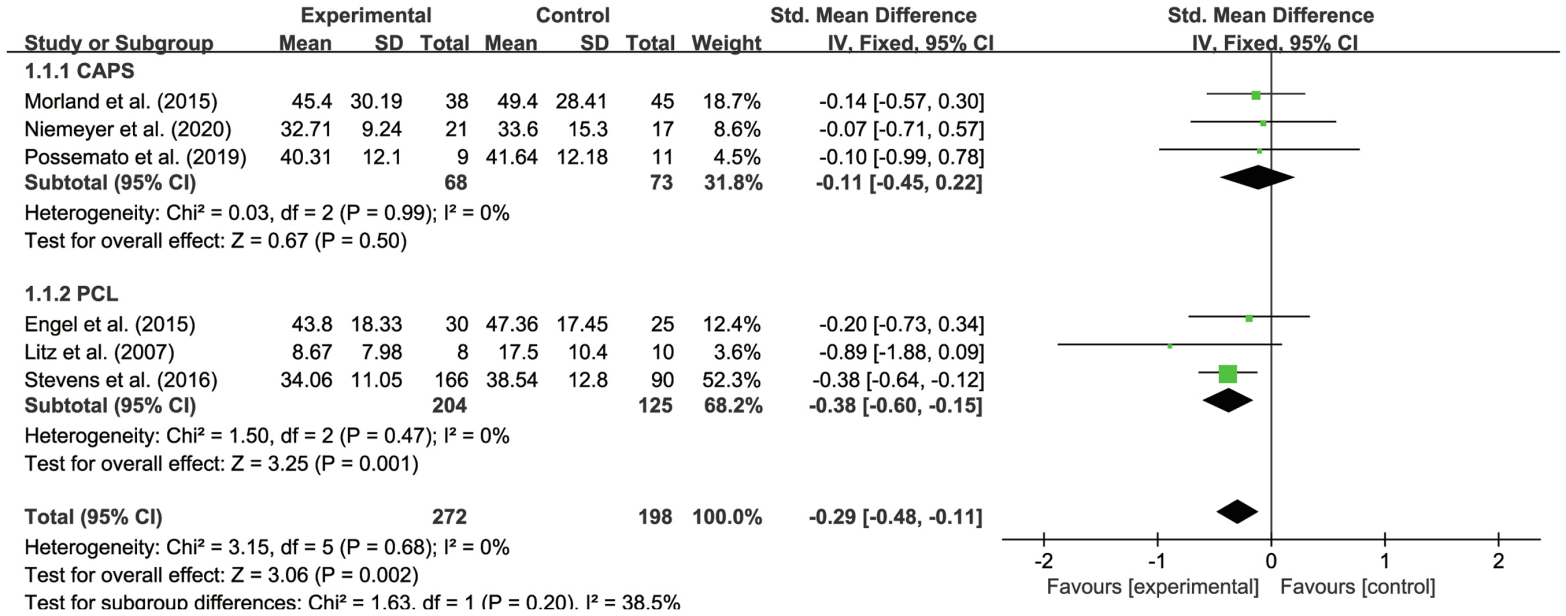
Forest plot of effects of IBI on PTSD with different instruments.

Concerning outcome measurements of PTSD, we split the instruments into two subgroups: CAPS (Morland et al., 2015; Possemato et al., 2019; Niemeyer et al., 2020) and PCL (Litz et al., 2007; Stevens et al., 2016; Engel et al., 2015). It was proven that IBI was more effective on PTSD measured with PCL (SMD=−0.38, 95% CI −0.60 to −0.15) than was PTSD evaluated with CAPS (SMD=−0.11, 95% CI −0.45 to 0.22). As shown in Figure 5, it was proved that the subgroups were not significantly different (*p*>0.05; *I*^2^ =38.5%), indicating that there was no heterogeneity between the groups. However, IBI has been found to be significantly effective on PTSD outcomes measured with PCL (*p*=0.001). IBI had no statistically significant effects on veterans’ PTSD outcome accessed with CAPS (*p*>0.05).

**Figure 6:**
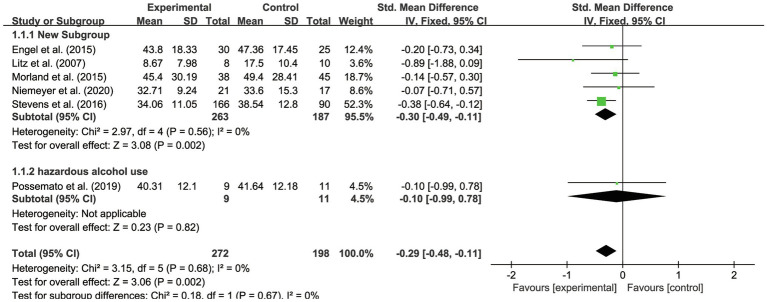
Forest plot of effects of IBI on veterans with different comorbidity.

In the six studies, the veterans with PTSD all had comorbidity. Five had a sample with veterans with depression and anxiety symptoms in addition to PTSD (Litz et al., 2007; Engel et al., 2015; Morland et al., 2015; Stevens et al., 2016; Niemeyer et al., 2020), and one study had a sample with veterans with comorbid PTSD and hazardous alcohol use (Possemato et al., 2019). IBI was shown more effective in veterans having depression and anxiety as comorbidities (SMD=−0.30, 95% CI −0.49 to −0.11) than in veterans with PTSD and hazardous alcohol use (SMD=−0.10, 95% CI −0.99 to 0.78). As presented in Figure 6, the subgroups were not significantly different (*p*>0.05; *I*^2^ =0%), proving that there was no heterogeneity between the two groups. IBI was found to work significantly on veterans with PTSD, depression, and anxiety (*p*<0.01) and have no statistically significant effects on veterans with PTSD and hazardous alcohol use (*p*>0.05).

### Results of Sensitivity Analysis

**Figure 7:**
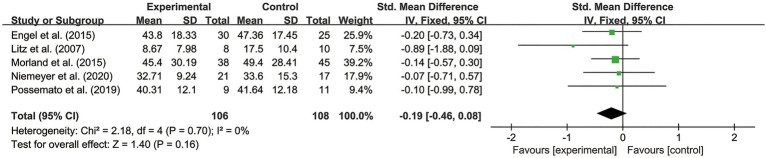
Forest plot of results without the study with the largest sample size.

In order to verify the stability of the results of the meta-analysis, the exclusion of a single study with a significantly large sample size was conducted to test its effects on the overall results. Based on the results shown in Table 4, of the six studies, the one by Stevens et al. (2016), an RCT from the United States with the largest sample size (166 participants in the experimental group and 90 participants in the control group) was eliminated. The sample size was much larger than that of other included studies. After excluding this study, the results (Figure 7) showed *p*>0.05. This suggests that the overall effect was not significant, which is inconsistent with the conclusion before the sensitivity analysis, indicating that the overall results of this combination were unstable.

**Figure 8:**
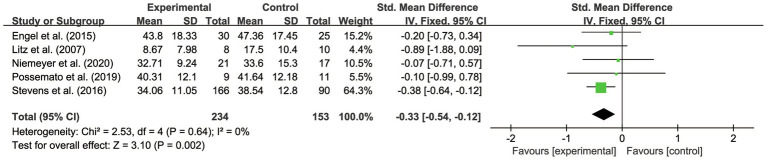
Forest plot of results without the study with female samples only.

**Figure 9:**
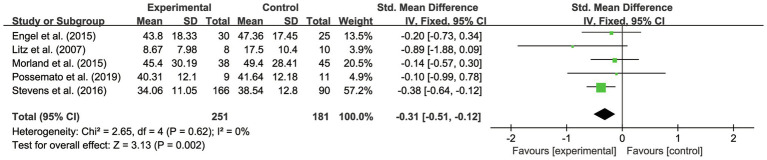
Forest plot of results without the study with male samples and different cultures.

The study by Morland et al. (2015) was an RCT in the United States with a sample of female veterans and female civilians. It was also reported as a high-risk study lacking reports on SD. After excluding this study (Figure 8), it showed that the overall effect was significant, which is consistent with the conclusion before the sensitivity analysis, suggesting that the overall results of this combination were stable. Similarly, Niemeyer et al. (2020) conducted an RCT with a sample of servicemen and male veterans in Germany. The trial was reported to be high risk in other biases. After excluding this study (Figure 9), it proved that the overall effect was significant, which is also consistent with the conclusion before the sensitivity analysis, showing that the overall results of this combination were stable.

## Discussion

### Principal Findings

Several systematic reviews and meta-analyses of IBI have been conducted on a reduction in symptoms of the mental health of caregivers, including depression, stress or distress, and anxiety (Guay et al., 2017; Hopwood et al., 2018; Sherifali et al., 2018; Zhao et al., 2019). However, less often discussed are the effects on veterans of PTSD. This is the first meta-analysis to include RCT studies, and it is targeted at reducing the symptoms of PTSD of veterans using IBI. We screened the research based on the inclusion and exclusion criteria. A total of six studies were included in the final analysis, and these were assessed with a low risk of bias. All were RCTs done with rigorous research and experimental design and provided high-qualified. The results of these studies reveal the beneficial effects of IBI on veterans with PTSD. A subgroup analysis was conducted to explore the interventions that were the most beneficial for veterans’ PTSD, the efficacy differences within diverse outcome measurements, and the differences of the effects on groups with particular comorbidity. The quality of evidence was examined according to the GRADE scores. The quality of evidence was high for the outcomes examined. The research in this review was almost low-risk, indicating the convincing quality of evidence. However, the literature had a common problem of lacking research details and quality reports.

In this meta-analysis, we proved that IBI was indeed beneficial in reducing the effects of PTSD on veterans. This is consistent with the review that IBI is effective in lessening the effects on anxiety and depression in caregivers of persons with disabilities (PwD; Zhao et al., 2019). One possibility is the advantages of technology. The new modality offered strengths over traditional treatment methods, including a reduction in traveling time and transportation costs and enhancement of access to treatment for persons with serious injuries, physical disabilities, or scheduling difficulties (Bose et al., 2001). The efficacy, feasibility, and acceptability of this new approach have proved preferable, according to a review on psychotherapy delivered through VTC among diverse patient populations (Backhaus et al., 2012). Moreover, the effectiveness could come from the recipients feeling safer and more comfortable when using online services in their own homes (Krupnick et al., 2017). All trials showed there were positive improvements by various interventions on veterans’ PTSD status to some degree. Considering sample size, test efficiency, rates of loss in follow-up, lack of follow-up, and the evolution of veterans’ illness (Melville et al., 2011; Zhao et al., 2019), we set a rule and chose the reported improved scores among several results of follow-ups. There was no follow-up in several studies (Stevens et al., 2016; Niemeyer et al., 2020). Also, the reduction of PTSD symptoms was greatest at 12-week follow-up but decreased over time. No benefits remained at 18-week follow-up (Engel et al., 2015). Another study found one significant enhancement at three-month follow-up (Morland et al., 2015). According to Litz et al. (2007), there was a great reduction in PTSD, and the veterans gained high functioning at 6months. Also, continuing improvements have been observed in post-treatment (12weeks) and follow-up (24weeks) in the trial by Possemato et al. (2019). Thus, we chose the reported means and SD of 12-week follow-up (Engel et al., 2015), 3-month follow-up (Morland et al., 2015), 6-month follow-up (Litz et al., 2007), and 24-week follow-up (Possemato et al., 2019).

Regarding the categories and components of IBI, several aspects of IBI interventions showed a promising reduction in PTSD symptoms for veterans. Subgroup analyses were conducted to determine which components were effective in supporting veterans with PTSD (Hopwood et al., 2018). According to the results, CBT with peer support has more positive effects on veterans’ PTSD than the other two kinds of IBI. This is highly consistent with one study finding that peer support helped patients manage physical health concerns effectively in peer navigation and support in self-management interventions (Cabassa et al., 2017). Supportive Accountability has clearly pointed out that interpersonal support improves the efficacy of eHealth interventions (Mohr et al., 2011). Only IBI based on CBT with peer support has been found to be significantly effective on PTSD outcome, which indicates that these two types of IBI might be not appropriate for veterans struggling with PTSD. It also shows that IBI is more effective on PTSD measured with PCL than was the outcome evaluated with CAPS. It might be that the study by Stevens et al. (2016) used the PCL-M scale especially for the military, which was more appropriate to measure veterans’ situations than was CAPS as a general scale to access PTSD status. In particular, this study has the largest sample (166) of the six studies, which might play a decisive role. This result suggests that IBI is effective in PTSD outcomes measured with PCL, meaning PCL might be a significantly appropriate instrument for testing the effects of IBI on veterans with PTSD. Similarly, IBI is shown more effective in veterans with depression and anxiety as comorbidity than in veterans with PTSD and hazardous alcohol use. This is in accordance with the research finding that online interventions reported a significant decrease in caregivers’ depressive symptoms and anxiety (Beauchamp et al., 2005; Hopwood et al., 2018). In particular, the internet proved an effective modality to deliver self-care for depression and PTSD (Christensen et al., 2002; Wagner et al., 2011). IBI has been found to work on veterans with PTSD and depression and anxiety only, suggesting that IBI might be significantly appropriate across veterans with comorbid PTSD and depression and anxiety.

Based on the results of sensitivity analysis, the sample size is a key element that weakens the stability of meta-analysis. It has also been discussed in other reviews that the sample size of studies was insufficient (Sherifali et al., 2018; Zhao et al., 2019). In this review, the studies by Litz et al. (2007) and Possemato et al. (2019) were both based on a small sample size (<20).

### Implications for Interventions and Researches

To develop IBI for veterans, some issues need to be addressed. The question of willingness of utilization is first. In one study, it was found out that only 30.9% of the veterans with probable PTSD were willing to use online interventions when faced with all seven kinds of e-Mental health services (Whealin et al., 2015). To solve this problem, familiar-sounding language and optimization of intervention should be used in the future, through which the veterans would feel more motivated to apply IBI (Murray et al., 2016). Based on the findings of subgroup analysis, intervention contents of IBI played a distinguished role in the reduction of veterans’ symptoms of PTSD. Among diverse contents provided by IBI, CBT with peer support offered the most meaningful support for veterans with PTSD, and more similar online services could be delivered accordingly. Secondly, computer literacy and access to the internet are factors in need of attention, and this is described as a digital divide in the literature (Bernhardt, 2000). This is necessary before participation in IBI. Finally, internet privacy and security are emphasized (Schaller et al., 2016), in order to avoid secondary injury of veterans.

This meta-analysis has demonstrated the demands for more and high-quality RCTs to access the effects of IBI on veterans. Based on the results of subgroup analysis, more details like instruments of outcomes and comorbidity of participants could be evaluated in the research design. Meanwhile, a sufficient sampling size of participants could be set to raise the quality of RCT researches. Future studies should describe more details of interventions to help us understand the elements of IBI that provide the most benefits. Future research should focus on the different types of militaries and indicate the content of IBI that is beneficial for the differentiated groups. Moreover, this review failed to distinguish the treatment trajectory in various stages of PTSD among veterans. All IBI in this review was broad for the course of the entire treatment process, ignoring the special needs during each stage of PTSD, which should be addressed by future studies.

### Strength and Limitations

This systematic review and meta-analysis have summarized the most relevant evidence in assessing the benefits of IBI on veterans’ PTSD outcomes. All six studies were published between 2007 and 2020, most published from 2015 to 2020, emphasizing the increasing interest in internet technologies to support veterans. This research is the most recent review and meta-analysis which examines the effects of IBI on PTSD outcomes of veterans, using the latest empirical studies and innovative subgroup analysis. In particular, it includes both Chinese and English studies, which take full account of cultural diversity.

However, three qualified research studies were dismissed without the full text in this meta-analysis. Also, only RCTs have been searched. Some of the trial protocols and reports have not been published. The lack of grey literature and limitation of language may lead to selection bias.

## Conclusion

The basis of evidence for IBI on veterans with PTSD remains limited. This meta-analysis is the first study to discuss the effectiveness of IBI on veterans. IBI has been proved as a positive modality to reduce PTSD symptoms among veterans. Moreover, it encourages us to focus on IBI with CBT with peer support for veterans, specific instruments for veterans’ PTSD, and veterans with comorbid depression and anxiety. Our research results highlight the promising potential of IBI to support veterans. It warrants further development and testing within more RCTs with high-quality and diverse cultural backgrounds.

## Data Availability Statement

The original contributions presented in the study are included in the article/Supplementary Material, further inquiries can be directed to the corresponding author.

## Author Contributions

YZ, ZB, and WW designed this meta-analysis. CW, ZF, LL, and SL searched the literature. CW, ZF, and YZ evaluated the risk of bias and conducted GRADE. CW completed the writing of Methods. ZF finished the writing of Findings. YZ and ZF wrote the Discussion. YZ, LL, and SL contributed to the Introduction and Literature Review. ZB and WW modified the manuscript. All authors contributed to the article and approved the submitted version.

## Funding

This study is supported by the National Social Science Foundation of China (20CSH086).

## Conflict of Interest

The authors declare that the research was conducted in the absence of any commercial or financial relationships that could be construed as a potential conflict of interest.

## Publisher’s Note

All claims expressed in this article are solely those of the authors and do not necessarily represent those of their affiliated organizations, or those of the publisher, the editors and the reviewers. Any product that may be evaluated in this article, or claim that may be made by its manufacturer, is not guaranteed or endorsed by the publisher.

## References

[ref1] Backhaus A. Agha Z. Maglione M. L. Repp A. Ross B. Zuest D. . (2012). Videoconferencing psychotherapy: a systematic review. Psychol. Serv. 9, 111–131. doi: 10.1037/a0027924, PMID: 22662727

[ref2] Barak A. Klein B. Proudfoot J. G. (2009). Defining internet-supported therapeutic interventions. Ann. Behav. Med. 38, 4–17. doi: 10.1007/s12160-009-9130-7, PMID: 19787305

[ref3] Beauchamp N. Irvine A. B. Seeley J. Johnson B. (2005). Worksite-based internet multimedia program for family caregivers of persons with dementia. The Gerontologist 45, 793–801. doi: 10.1093/geront/45.6.793, PMID: 16326661

[ref4] Belsher B. E. Kuhn E. Maron D. Prins A. Cueva D. Fast E. . (2015). A preliminary study of an internet-based intervention for OEF/OIF veterans presenting for VA specialty PTSD care. J. Trauma. Stress. 28, 153–156. doi: 10.1002/jts.21994, PMID: 25864506

[ref5] Bernhardt J. M. (2000). Health education and the digital divide: building bridges and filling chasms. Health Educ. Res. 15, 527–531. doi: 10.1093/her/15.5.527, PMID: 11184212

[ref6] Bose U. McLaren P. Riley A. Mohammedali A. (2001). The use of telepsychiatry in the brief counselling of non-psychotic patients from an inner-London general practice. J. Telemed. Telecare 7(Suppl 1), 8–10. doi: 10.1177/1357633X010070S103, PMID: 11576473

[ref7] Bush N. E. Bosmajian C. P. Fairall J. M. Mccann R. A. Ciulla R. P. (2011). afterdeployment.org: A web-based multimedia wellness resource for the postdeployment military community. Prof. Psychol. Res. Pr. 42, 455–462. doi: 10.1037/a0025038

[ref8] Cabassa L. J. Camacho D. Velez-Grau C. M. Stefancic A. (2017). Peer-based health interventions for people with serious mental illness: A systematic literature review. J. Psychiatr. Res. 84, 80–89. doi: 10.1016/j.jpsychires.2016.09.021, PMID: 27701013

[ref9] Christensen H. Griffiths K. M. Korten A. (2002). Web-based cognitive behavior therapy: analysis of site usage and changes in depression and anxiety scores. J. Med. Internet Res. 4:e3. doi: 10.2196/jmir.4.1.e3, PMID: 11956035 PMC1761927

[ref10] Colgan D. D. Christopher M. Michael P. Wahbeh H. (2016). The body scan and mindful breathing among veterans with PTSD: type of intervention moderates the relationship between changes in mindfulness and post-treatment depression. Mindfulness 7, 372–383. doi: 10.1007/s12671-015-0453-0, PMID: 32863982 PMC7451147

[ref11] Engel C. C. Litz B. Magruder K. M. Harper E. Gore K. Stein N. . (2015). Delivery of self training and education for stressful situations (DESTRESS-PC): a randomized trial of nurse assisted online self-management for PTSD in primary care. Gen. Hosp. Psychiatry 37, 323–328. doi: 10.1016/j.genhosppsych.2015.04.007, PMID: 25929985 PMC4762212

[ref12] Grade Working Group (2004). Grading quality of evidence and strength of recommendations. Br. Med. J. 328, 1490. doi: 10.1136/bmj.328.7454.1490, PMID: 15205295 PMC428525

[ref13] Guay C. Auger C. Demers L. Mortenson W. B. Ahmed S. (2017). Components and outcomes of internet-based interventions for caregivers of older adults: systematic review. J. Med. Internet Res. 19:e313. doi: 10.2196/jmir.7896, PMID: 28928109 PMC5627044

[ref14] Haagen J. F. Smid G. E. Knipscheer J. W. Kleber R. J. (2015). The efficacy of recommended treatments for veterans with PTSD: A metaregression analysis. Clin. Psychol. Rev. 40, 184–194. doi: 10.1016/j.cpr.2015.06.008, PMID: 26164548

[ref15] Hamblen J. L. Grubaugh A. L. Davidson T. M. Borkman A. L. Bunnell B. E. Ruggiero K. J. (2019). An online peer educational campaign to reduce stigma and improve help seeking in veterans with posttraumatic stress disorder. Telemed. J. E Health 25, 41–47. doi: 10.1089/tmj.2017.0305, PMID: 29746232

[ref16] Harris J. I. Erbes C. R. Engdahl B. E. Thuras P. Murray-Swank N. Grace D. . (2011). The effectiveness of a trauma focused spiritually integrated intervention for veterans exposed to trauma. J. Clin. Psychol. 67, 425–438. doi: 10.1002/jclp.20777, PMID: 21294116

[ref17] Higgins J. P. Altman D. G. Gotzsche P. C. Juni P. Moher D. Oxman A. D. . (2011). The Cochrane collaboration's tool for assessing risk of bias in randomised trials. Br. Med. J. 343, d5928. doi: 10.1136/bmj.d5928, PMID: 22008217 PMC3196245

[ref18] Higgins J. P. T. Thompson S. G. Deeks J. J. Altman D. G. (2003). Measuring inconsistency in meta-analysis. Br. Med. J. 327, 557–560. doi: 10.1136/bmj.327.7414.557, PMID: 12958120 PMC192859

[ref19] Hopwood J. Walker N. Mcdonagh L. Rait G. Walters K. Iliffe S. . (2018). Internet-based interventions aimed at supporting family caregivers of people with dementia: systematic review. J. Med. Internet Res. 20:e216. doi: 10.2196/jmir.9548, PMID: 29895512 PMC6019848

[ref20] Kearney D. J. McDermott K. Malte C. Martinez M. Simpson T. L. (2012). Association of participation in a mindfulness program with measures of PTSD, depression and quality of life in a veteran sample. J. Clin. Psychol. 68, 101–116. doi: 10.1002/jclp.20853, PMID: 22125187

[ref21] King A. P. Erickson T. M. Giardino N. D. Favorite T. Rauch S. A. Robinson E. . (2013). A pilot study of group mindfulness-based cognitive therapy (MBCT) for combat veterans with posttraumatic stress disorder (PTSD). Depress. Anxiety 30, 638–645. doi: 10.1002/da.22104, PMID: 23596092 PMC4373594

[ref22] Koven S. G. (2018). Veteran treatments: PTSD interventions. Healthcare 6, 94. doi: 10.3390/healthcare6030094, PMID: 30082634 PMC6164350

[ref23] Krupnick J. L. Green B. L. Amdur R. Alaoui A. Belouali A. Roberge E. . (2017). An Internet-based writing intervention for PTSD in veterans: a feasibility and pilot effectiveness trial. Psychol. Trauma 9, 461–470. doi: 10.1037/tra0000176, PMID: 27607767

[ref24] Kulesza M. Pedersen E. R. Corrigan P. W. Marshall G. N. (2015). Help-seeking stigma and mental health treatment seeking among young adult veterans. Mil. Behav. Health 3, 230–239. doi: 10.1080/21635781.2015.1055866, PMID: 26664795 PMC4672863

[ref25] Litz B. T. Engel C. C. Bryant R. A. Papa A. (2007). A randomized, controlled proof-of-concept trial of an internet-based, therapist-assisted self-management treatment for posttraumatic stress disorder. Am. J. Psychiatr. 164, 1676–1684. doi: 10.1176/appi.ajp.2007.06122057, PMID: 17974932

[ref26] McLean C. P. Foa E. B. (2013). Dissemination and implementation of prolonged exposure therapy for posttraumatic stress disorder. J. Anxiety Disord. 27, 788–792. doi: 10.1016/j.janxdis.2013.03.004, PMID: 23602350

[ref27] Melville K. M. Casey L. M. Kavanagh D. J. (2011). Dropout from internet-based treatment for psychological disorders. Br. J. Clin. Psychol. 49, 455–471. doi: 10.1348/014466509X472138, PMID: 19799804

[ref28] Moher D. (2010). Corrigendum to: preferred reporting items for systematic reviews and meta-analyses: The PRISMA statement. Int. J. Surg. 8, 658–341. doi: 10.1016/j.ijsu.2010.07.299 20171303

[ref29] Mohr D. C. Cuijpers P. Lehman K. (2011). Supportive accountability: A model for providing human support to enhance adherence to ehealth interventions. J. Med. Internet Res. 13:e30. doi: 10.2196/jmir.1602, PMID: 21393123 PMC3221353

[ref30] Monson C. M. Taft C. T. Fredman S. J. (2009). Military-related PTSD and intimate relationships: from description to theory-driven research and intervention development. Clin. Psychol. Rev. 29, 707–714. doi: 10.1016/j.cpr.2009.09.002, PMID: 19781836 PMC2783889

[ref31] Morland L. A. Mackintosh M. A. Rosen C. S. Willis E. Resick P. Chard K. . (2015). Telemedicine versus in-person delivery of cognitive processing therapy for women with posttraumatic stress disorder: A randomized noninferiority trial. Depress. Anxiety 32, 811–820. doi: 10.1002/da.22397, PMID: 26243685

[ref32] Murray E. Hekler E. B. Andersson G. Collins L. M. Doherty A. Hollis C. . (2016). Evaluating digital health interventions: key questions and approaches. Am. J. Prev. Med. 51, 843–851. doi: 10.1016/j.amepre.2016.06.008, PMID: 27745684 PMC5324832

[ref33] Niemeyer H. Knaevelsrud C. Schumacher S. Engel S. Kuester A. Burchert S. . (2020). Evaluation of an internet-based intervention for service members of the German armed forces with deployment-related posttraumatic stress symptoms. BMC Psychiatry 20:205. doi: 10.1186/s12888-020-02595-z, PMID: 32375754 PMC7204035

[ref34] Parish M. B. Apperson M. Yellowlees P. M. (2014). Engaging U.S. veterans with PTSD in online therapy. Psychiatric Services 65:697. doi: 10.1176/appi.ps.650501, PMID: 24788736

[ref35] Possemato K. Johnson E. M. Emery J. B. Wade M. Maisto S. A. (2019). A pilot study comparing peer supported web-based CBT to self-managed web CBT for primary care veterans with PTSD and hazardous alcohol use. Psychiatr. Rehabil. J. 42, 305–313. doi: 10.1037/prj0000334, PMID: 30489140 PMC6541543

[ref36] Possemato K. Ouimette P. Knowlton P. (2011). A brief self-guided telehealth intervention for post-traumatic stress disorder in combat veterans: a pilot study. J. Telemed. Telecare 17, 245–250. doi: 10.1258/jtt.2011.100909, PMID: 21636687

[ref37] Reddy S. Dick A. M. Gerber M. R. Mitchell K. (2014). The effect of a yoga intervention on alcohol and drug abuse risk in veteran and civilian women with posttraumatic stress disorder. J. Altern. Complement. Med. 20, 750–756. doi: 10.1089/acm.2014.0014, PMID: 25211372 PMC4195227

[ref38] Rodriguez K. E. LaFollette M. R. Hediger K. Ogata N. O'Haire M. E. (2020). Defining the PTSD service dog intervention: perceived importance, usage, and symptom specificity of psychiatric service dogs for military veterans. Front. Psychol. 11:1638. doi: 10.3389/fpsyg.2020.01638, PMID: 32849004 PMC7396623

[ref39] Sautter F. J. Glynn S. M. Cretu J. B. Senturk D. Vaught A. S. (2015). Efficacy of structured approach therapy in reducing PTSD in returning veterans: A randomized clinical trial. Psychol. Serv. 12, 199–212. doi: 10.1037/ser0000032, PMID: 26213789

[ref40] Schaller S. Marinova-Schmidt V. Setzer M. Kondylakis H. Griebel L. Sedlmayr M. . (2016). Usefulness of a tailored ehealth service for informal caregivers and professionals in the dementia treatment and care setting: The ehealth monitor dementia portal. JMIR Res. Protoc. 5:e47. doi: 10.2196/resprot.4354, PMID: 27050401 PMC4822652

[ref41] Sherifali D. Ali M. U. Ploeg J. Markle-Reid M. Valaitis R. Bartholomew A. . (2018). Impact of internet-based interventions on caregiver mental health: systematic review and meta-analysis. J. Med. Internet Res. 20:e10668. doi: 10.2196/10668, PMID: 29970358 PMC6053616

[ref42] Simblett S. Birch J. Matcham F. Yaguez L. Morris R. (2017). A systematic review and meta-analysis of e-mental health interventions to treat symptoms of posttraumatic stress. JMIR Ment. Health 4:e14. doi: 10.2196/mental.5558, PMID: 28526672 PMC5451639

[ref43] Stevens N. R. Holmgreen L. Walt L. Gengler R. Hobfoll S. E. (2016). Web-based trauma intervention for veterans has physical health payoff in randomized trial. Psychol. Trauma Theory Res. Pract. Policy 9(Suppl 1), 42–50. doi: 10.1037/tra0000184, PMID: 27657979

[ref44] Turgoose D. Murphy D. (2019). A systematic review of interventions for supporting partners of military veterans with PTSD. J. Mil. Veteran Fam. Health 5, 195–208. doi: 10.3138/jmvfh.2018-0035

[ref45] Wagner B. Schulz W. Knaevelsrud C. (2011). Efficacy of an internet-based intervention for posttraumatic stress disorder in Iraq: A pilot study. Psychiatry Res. 195, 85–88. doi: 10.1016/j.psychres.2011.07.026, PMID: 21813187

[ref46] Whealin J. M. Seibert-Hatalsky L. A. Howell J. W. Tsai J. (2015). E-mental health preferences of veterans with and without probable posttraumatic stress disorder. J. Rehabil. Res. Dev. 52, 725–738. doi: 10.1682/JRRD.2014.04.0113, PMID: 26562090

[ref47] Williamson V. Stevelink S. A. M. Greenberg K. Greenberg N. (2018). Prevalence of mental health disorders in elderly U.S. military veterans: A meta-analysis and systematic review. Am. J. Geriatr. Psychiatr. 26, 534–545. doi: 10.1016/j.jagp.2017.11.001, PMID: 29221697

[ref48] Zhao Y. Feng H. Hu M. Hu H. Li H. Ning H. . (2019). Web-based interventions to improve mental health in home caregivers of people with dementia: meta-analysis. J. Med. Internet Res. 21:e13415. doi: 10.2196/13415, PMID: 31066680 PMC6526687

